# Heart Failure With Preserved Ejection Fraction: An Evolving Understanding

**DOI:** 10.7759/cureus.46152

**Published:** 2023-09-28

**Authors:** Sunanda Tah, Melissa Valderrama, Maham Afzal, Javed Iqbal, Aisha Farooq, Muhammad Ali Lak, Karol Gostomczyk, Elhama Jami, Mahendra Kumar, Akshay Sundaram, Mouhammad Sharifa, Mustafa Arain

**Affiliations:** 1 Surgery, Beckley Appalachian Regional Healthcare (ARH) Hospital, Beckley, USA; 2 Surgery, Saint James School of Medicine, Arnos Vale, VCT; 3 Medicine, Pontificia Universidad Javeriana, Cali, COL; 4 Medicine, Fatima Jinnah Medical University, Lahore, PAK; 5 Neurosurgery, Mayo Hospital, Lahore, PAK; 6 Internal Medicine, Dr. Ruth Pfau Hospital, Karachi, PAK; 7 Internal Medicine, Combined Military Hospital, Lahore, PAK; 8 Medicine, Collegium Medicum Nicolaus Copernicus University, Bydgoszcz, POL; 9 Internal Medicine, Herat Regional Hospital, Herat, AFG; 10 Medicine, Sardar Patel Medical College, Bikaner, IND; 11 Internal Medicine, Kasturba Medical College, Manipal, IND; 12 Medicine, University of Aleppo, Aleppo, SYR; 13 Internal Medicine, Civil Hospital Karachi, Karachi, PAK

**Keywords:** diagnosis, left ventricular filling pressure, treatment, preserved ejection fraction, hear failure

## Abstract

Heart failure (HF) with preserved ejection fraction (HFpEF) is a clinical syndrome in which patients have signs and symptoms of HF due to high left ventricular (LV) filling pressure despite normal or near normal LV ejection fraction. It is more common than HF with reduced ejection fraction (HFrEF), and its diagnosis and treatment are more challenging than HFrEF. Although hypertension is the primary risk factor, coronary artery disease and other comorbidities, such as atrial fibrillation (AF), diabetes, chronic kidney disease (CKD), and obesity, also play an essential role in its formation. This review summarizes current knowledge about HFpEF, its pathophysiology, clinical presentation, diagnostic challenges, current treatments, and promising novel treatments. It is essential to continue to be updated on the latest treatments for HFpEF so that patients always receive the most therapeutic treatments. The use of GnRH agonists in the management of HFpEF, infusion of Apo a-I nanoparticle, low-level transcutaneous vagal stimulation (LLTS), and estrogen only in post-menopausal women are promising strategies to prevent diastolic dysfunction and HFpEF; however, there is still no proven curative treatment for HFpEF yet.

## Introduction and background

A significant problem for worldwide public health is heart failure (HF) with preserved ejection fraction (HFpEF) [1,2]. HFpEF is a clinical condition that develops when the heart cannot pump blood effectively without an elevated cardiac filling pressure [3]. The phrase "HF with normal ejection fraction" has changed to the more recent concept of HFpEF [4] with the publication of a consensus statement by the Heart Failure Association (HFA) of the European Society of Cardiology (ESC) in 2007 [5].

Due to variations in diagnostic criteria and research populations, recent investigations have indicated that the prevalence of HFpEF ranges substantially from 13% to 74% [6-8]. The prevalence of HFpEF has generally increased yearly and now accounts for 50% of all HF cases [6]. According to research on consecutive patients, the prevalence of HFpEF increased every year from 1987 to 2001, and in the three consecutive five-year study periods, the average prevalence climbed from 38% to 47% to 54% [1]. Growing emphasis has been given to the high prevalence and poor prognosis of HFpEF. Age, hypertension, and the existence of ischemic heart disease are some of the risk factors for HFpEF that have recently been found by research [9-11]. It should be noted that most studies on the prevalence of HFpEF have been conducted in select populations, such as the elderly, inpatients, or outpatients [12-15]. Relatively few studies have been done on a general population.

It should be noted that the prevalence of HFpEF in the community rose with age and was greater in women; the reported age- and sex-specific prevalence increased from 0% (men) and 1% (women) in the age group of 25-49 years to around 4-6% in males and 8-10% in women for people 80 years and older [16].

There are a few gaps in our understanding of the epidemiology of HFpEF that should be investigated in the future. Different investigations of HFpEF use different diagnostic cut-points to establish a normal left ventricular ejection fraction (LVEF), with ESC recommendations favoring a 50% threshold [4]. The prevalence of HFpEF would decline if a higher cut-point (55%) was used to define normal LVEF.

Other information gaps concern the prevalence of HFpEF internationally (outside of the United States and Europe) and the variance in the disease burden by ethnicity. Future studies including multi-ethnic samples are required, as recent data suggest that Afro-Caribbeans may be more likely to suffer from diastolic dysfunction [17].

Additional research is required to determine the factors that raise the risk for HFpEF in women because it is generally known that women are more likely to have the condition [18].

This should be further researched because it is unknown whether HFpEF aggregates within families or if parental HFpEF increases the risk of the condition in the offspring [19]. In addition, given the high rates of obesity, dyslipidemia, and diabetes mellitus in patients with HFpEF, further research is necessary to determine how metabolic abnormalities, such as metabolic syndrome, contribute to the disease's increasing burden.

This review aims to comprehensively present the current understanding and knowledge regarding the pathogenesis, epidemiology, clinical presentation, and management of HFpEF, as well as thoroughly analyze information gaps, controversies, and challenges pertaining to this disease.

## Review

Pathophysiology of HFpEF

Diastolic Dysfunction and Its Role in HFpEF

HFpEF is a syndrome with a variety of underlying causes and numerous alterations in physiological functions that add to the complexity of the illness. The left ventricular (LV) diastolic dysfunction plays a fundamental role in this illness [20,21]. This dysfunction entails an increase in vesicular-elastic stiffness, a decrease in relaxation efficiency, or both [22,23], resulting in increased filling pressures during rest or activity that ultimately causes symptomatic HF [24].

Initial observational studies on HFpEF revealed a common occurrence of concentric hypertrophy of the left ventricle alongside a normal chamber size [25,26]. However, subsequent population-based investigations have shown that many HFpEF patients exhibit either concentric remodeling without hypertrophy or even maintain a normal LV geometry [27-29]. Cardiomyocytes in HFpEF appear thicker and have a less elongated shape compared to those observed in HF with reduced ejection fraction (HFrEF) [30]. Additionally, there is an increased collagen content in comparison to control populations [31].

Myocardial Fibrosis and Its Impact on Ventricular Compliance

The concept of diastolic function encompasses both an active pressure decay process (relaxation) occurring in early diastole, linked to myofilament separation and calcium reabsorption, as well as a "passive" stiffness associated with viscoelastic attributes influenced by mechanical shifts ranging from the sarcomere to the extracellular matrix, chamber, and pericardium [32,33]. High LV myocardial diastolic stiffness, as typically observed in HFpEF, arises from both myocardial fibrosis and reduced cardiomyocyte and myocardial distensibility. These observations have been directly measured in cardiomyocytes obtained from LV endomyocardial biopsies and myocardial muscle samples from LV epicardial biopsies [34]. Myocardial fibrosis is often identified through an elevated collagen content in the left ventricle or septal myocardium of HFpEF patients [35].

These changes result from specific modifications in the transcriptional and post-translational aspects of a significant sarcomeric protein known as titin [36-39]. The sarcomere macromolecule titin plays a central role in diastolic relaxation. It exists in N2B and N2A isoforms, with a higher proportion of N2B isoforms in HFpEF [40]. The phosphorylation of titin, which is regulated by the cGMP-protein kinase G-dependent pathway, influences diastolic tone and ventricular function [41].

Alterations in fibrillar collagen are closely tied to diastolic dysfunction. Conditions such as hypertension and aging, which are associated with diastolic dysfunction, often involve fibrosis [41]. This fibrosis is linked to increased oxidative stress and the presence of profibrotic cytokines [41]. Collagen degradation changes, involving matrix metalloproteases (MMPs) and tissue inhibitors of MMPs (TIMPs), contribute to LV remodeling [42]. Altered MMP-1 activity can lead to excessive collagen deposition and subsequent diastolic dysfunction [42]. Elevations in MMP-2 and MMP-9 or reductions in TIMP-1 are observed in asymptomatic diastolic dysfunction and diastolic HF [43]. The severity of collagen turnover correlates with the extent of diastolic dysfunction [44]. In systemic sclerosis, TIMP-1 levels are related to diastolic dysfunction and remodeling of the LV matrix [44]. Cardiac myosin binding protein C (cMyBP-C) also plays a crucial role in cross-bridge kinetics. Mutations in cMyBP-C can induce diastolic dysfunction, and its phosphorylation by protein kinase A affects cross-bridge turnover rates [45]. Recent research suggests that oxidative S-glutathionylation of cMyBP-C is associated with impaired relaxation, possibly due to increased myofilament Ca2+ sensitivity [46].

Impaired Endothelial Function and Vascular Contributions to HFpEF

Approximately 30% of HFpEF patients exhibit endothelium-dependent coronary microvascular dysfunction, while over 30% display endothelium-independent dysfunction seen as reduced coronary flow reserve [47]. HFpEF patients also experience vascular-ventricular stiffness, linked to decreased exercise capacity [48]. Increased cardiac afterload within this stiffness context raises arterial blood pressure, impairing diastolic relaxation and elevating filling pressures during exercise [49]. Mechanisms behind arterial elastance changes during HFpEF, tied to bioavailability and responses to vasoactive molecules like ET-1 and NO, remain partly unexplored [50,51].

Microvascular rarefaction, evidenced by diminished myocardial microvascular density, contributes to cardiac perfusion failure in HFpEF, reducing myocardial oxygen delivery [52,53]. This rarefaction raises coronary microvascular resistance, thus decreasing cardiac perfusion and potentially driving the progressive decline in cardiac function [54]. Arterial rarefaction and inadequate angiogenesis, part of microvascular/macrovascular dysfunction, can decrease myocardial oxygen supply [55]. Aortic stiffness and altered endothelial function contribute to diastolic dysfunction, while macrovascular stiffness relates to ventricular elastance reduction [56,57]. Notably, macrovascular dysfunction might stem from primary microvascular alterations [58]. This parallels observations of endothelial dysfunction in microvasculature without overt changes in conductance vessels in HFpEF models [59].

Inflammatory Mechanisms and Their Involvement in HFpEF

Several studies shed light on the cardioprotective impact of NO/sGC/cGMP/PKG signaling [60]. Endothelial NO synthase (eNOS) enhancer AVE9488 improves cardiac remodeling post-MI and influences platelet NO availability and hyperactivity in HF [61-62]. In senescence-accelerated-prone mice, a high-salt, high-fat diet induces HFpEF characterized by endothelial cell dysfunction and fibrosis, highlighting the role of diet-induced endothelial cell dysfunction in HFpEF [63]. Increased insulin-like growth factor-1 (IGF-1) activity mitigates age-related endothelial progenitor cell dysfunction [64]. Myeloperoxidase, released from activated neutrophils, modulates vascular inflammation and NO bioavailability [65], linking it to HF post-ischemic injury [66], atrial structural remodeling, and atrial fibrillation (AF) risk [67].

Inflammation significantly influences the development of HFpEF. This has been demonstrated through swine models with comorbidities, namely, arterial hypertension, diabetes, and hypercholesterolemia [68]. Diabetes, an HF risk factor, includes increased inflammation, driven by elevated interleukin (IL-1β, IL-6), intercellular adhesion molecule-1 (ICAM-1), and vascular cell adhesion molecule-1 (VCAM-1), and decreased activity of the collagen-degrading MMP [69,70]. Macrophages and monocytes influence ischemic injury-triggered ventricular remodeling [71,72]. Myocardial ischemia elicits three phases: acute inflammation, healing, and chronic inflammation. Ly6Chigh monocytes dominate the acute inflammatory phase, recruiting M1 macrophages and promoting inflammation [73]. Cardiac macrophages transition to M2, promoting repair [74]. Neutrophils contribute to macrophage M2 polarization [74]. The healing phase, driven by Ly6Clow monocytes, leads to fibroblast activation and proliferation [75]. Chronic inflammation exhibits elevated T-lymphocytes and M1 macrophages, linked to HF development [76,77].

Diagnostic challenges

Limitations of Current Diagnostic Criteria for HFpEF

The diagnostic criteria for HFpEF differ significantly among various society guidelines primarily because there is no consensus on how to define HFpEF. This lack of agreement is attributed to an incomplete understanding of the disease's pathobiology, the diversity in its presentation, and its natural progression over time [78].

However, the majority of the studies have defined HFpEF as the appearance of physical manifestations of HF, an LVEF that is 50 or more, elimination of "HFpEF mimickers," such as infiltrative cardiomyopathies and indications of heightened LV filling pressure or non-invasive markers (elevated E:e ratio, increased left atrial volume, elevated natriuretic peptides (NPs)) [5,79].

Hospitalized HFpEF (congestion at rest): The diagnosis of this condition is dependent on the classic signs and symptoms, as well as the use of NPs and imaging techniques such as chest radiography and echocardiography. However, LV hypertrophy may not be present in three out of four individuals [80-83], and NPs may appear normal in around 29% of obese patients, even if they have clinically overt HF [84]. In cases where the diagnosis is ambiguous, right heart catheterization can provide further clarification, although it is not routinely recommended [85].

Exercise intolerance HFpEF: Diagnosing this condition is complex when congestion is not present at rest. Physical examination results may or may not be normal in the case of normal LV filling pressures at rest [86-87]. For patients without breathlessness or evident signs of increased LV filling pressures at rest, further tests like exercise echocardiography or invasive exercise hemodynamics are necessary to uncover any underlying abnormal diastolic reserve [86-88]. However, imaging studies during exercise are prone to human error. Other factors, such as AF and structural heart disease identified through echocardiography, can help in the diagnosis. It is worth noting that NPs are often not elevated if rest congestion is absent [87].

Multiple randomized controlled trials exploring HFpEF treatments have adopted lower LVEF thresholds (40-45%) to increase the occurrence of events, thereby achieving the necessary statistical strength to prove therapy efficacy. Individuals with HF mid-range ejection fraction (HFmrEF) exhibit comparable underlying mechanisms and responsiveness to treatment as those with HF reduced ejection fraction (HFrEF). Consequently, involving HFmrEF patients in HFpEF trials supports the evaluation of treatments that have proven beneficial in HFrEF cases [89-90].

Two diagnostic approaches for HFpEF, namely the H2FPEF score [91] and the ESC HFA-PEFF algorithm [5], combine clinical features and diagnostic measures to differentiate between HFpEF and non-cardiac dyspnea. Both methods effectively pinpoint individuals at increased risk of HF events. However, when applied to diverse populations, there is some overlap between high scores in H2FPEF and HFA-PEFF [92]. These scoring systems perform reasonably, with the area under the curve ranging from 0.73 to 0.74, as validated against invasive hemodynamics [92]. It's important to note that both methods misclassify up to 23% of patients, particularly those with low scores but meeting invasive HFpEF criteria. Hence, a low score doesn't entirely rule out HFpEF possibility [92]. Additionally, a significant portion of patients fall into the intermediate probability range, necessitating further testing [2].

Role of Advanced Imaging Techniques in HFpEF

Functional imaging techniques, particularly cardiovascular magnetic resonance (CMR), have emerged as important tools for assessing the complex pathophysiology of HFpEF at a more detailed level. Conventional diagnostic methods often fail to capture the subtle changes associated with HFpEF, making functional imaging an essential complement. CMR provides high-resolution imaging, allowing accurate assessment of atrial and ventricular volumes, ejection fraction, and diastolic function. Pathologic changes such as left atrial and LV hypertrophy have been associated with HFpEF. Additionally, CMR can provide insights into myocardial stiffness evidenced by changes in blood flow patterns and myocardial strain [92-94].

One novel CMR technique, known as "4D-flow," enables the quantification of three-dimensional blood flow velocities throughout the cardiac cycle which allows the study of filling dynamics in HFpEF patients, offering a further understanding of the fluid-dynamical alterations in the condition [95-96]. Recent advancements in AI algorithms can help interpret these alterations speedily [97].

Metabolic imaging methods, such as phosphorus-31 cardiac magnetic resonance spectroscopy, provide valuable such as decreased phosphocreatine/adenosine triphosphate ratios in HFpEF patients, indicating altered myocardial energetics [98]. Moreover, 11C-acetate PET imaging has shown changes in cardiac oxidative metabolism during stress, reflecting the reduced exercise capacity of the HFpEF heart [99-100].

Biomarkers and their potential to aid in early diagnosis of HFpEF

NPs

Levels of NPs are increased in patients with HFpEF, reflecting the severity of cardiac structural and functional irregularities like LV hypertrophy, fibrosis, and impaired diastolic function [101-102]. Therefore, measuring these levels plays an important role in the diagnostic process for HF [2,103-104]. NP levels are moderately higher in HFpEF compared to HFrEF, yet a specific threshold to accurately differentiate between the two conditions has not been established [104-105]. A comprehensive review of 51 studies indicated that NPs exhibit reasonable diagnostic accuracy in identifying HFpEF, with an AUC of 0.80 and a 95% CI ranging from 0.73 to 0.87 [106].

The presence of other medical conditions has an impact on NP levels in both HFpEF and HFrEF, a particularly pertinent consideration given the higher prevalence of non-cardiac comorbidities in HFpEF. A number of conditions correlate with elevated NPs, including chronic obstructive pulmonary disease (COPD), AF, kidney disease, and diabetic ketosis, whereas obese patients may exhibit lower NP levels [107-109]. Additionally, advancing age is linked to higher NP concentrations [107,110].

Troponin

In individuals with HF, increased troponin levels generally correspond to the severity of HF, particularly in acute situations. However, elevated troponin levels have also been observed in chronic HF, possibly due to factors like inflammation, neurohormonal activation, myocardial strain, hypoxia, and cytotoxicity [111].

Numerous studies have examined the predictive significance of troponin testing in patients admitted for HFpEF. In a group of 500 patients with an ejection fraction of 40% or more, troponin T (TnT) levels were directly linked to serum creatinine and the severity of symptoms. Furthermore, they independently predicted both all-cause mortality and the need for rehospitalization due to HF [112].

Other Biomarkers

HFpEF patients exhibit higher levels of certain biomolecules such as soluble suppression of tumorigenesis 2 (sST2), hs-CRP, and cystatin-C, TIMPs, N-terminal propeptide of type III procollagen (PIIINP), homocysteine, and resistin. The most extensively studied biomarkers in HFpEF include sST2, galectin-3 (Gal-3), and growth differentiation factor 15 (GDF-15). Whereas, in HFrEF, increased levels of NT-proBNP, hs-TnT, inflammatory biomarkers like pentraxin-3, tumor necrosis factor-alpha (TNF-a), IL-6, and IL-10 are more prominent [113,114].

Clinical presentation and comorbidities

Risk Factors and Comorbidities in HFpEF

Cardiovascular risk factors play a significant role in HFpEF development and progression. Hypertension is a major risk factor, with a prevalence ranging from 55% to 90% among HFpEF patients. Proper blood pressure control is essential in managing hypertensive patients with HFpEF as it affects LV hypertrophy, fibrosis, and diastolic dysfunction [115-117].

AF is another well-known risk factor and prognostic indicator in HFpEF. AF and HFpEF exhibit mutual causation; AF can lead to HFpEF and vice versa. AF complicates HFpEF diagnosis due to shared clinical manifestations, and it is an independent prognostic factor for mortality and HF hospitalization [118-120].

Diabetes is prevalent in approximately one-third of HFpEF patients and contributes to oxidative stress and endothelial dysfunction. Proper management of diabetes is crucial in HFpEF patients to address its impact on pathophysiology [121-122].

Obesity is a major risk factor for incident HFpEF, with nearly 80% of HFpEF patients being overweight or obese. Obesity leads to inflammation and altered cardiac hemodynamics, which require further investigation [123].

Chronic kidney disease (CKD) is another significant risk factor for HFpEF, affecting around one-third of HFpEF patients. CKD worsens HFpEF outcomes, and its coexistence with HFpEF, known as cardiorenal syndrome, requires careful management [124-125].

Other comorbidities impacting HFpEF outcomes include COPD, iron deficiency, and anemia [79,126-128].

Detecting specific secondary causes of HFpEF, such as infiltrative restrictive cardiomyopathy and restrictive cardiomyopathy due to various conditions, is crucial for effective treatment [129-130].

HFpEF by Age

Traditionally, HFpEF has been associated with aging, and studies demonstrate an age-dependent increase in its prevalence. However, recent research indicates that younger individuals, particularly in Asia, are also affected, with up to 15% of HFpEF patients being young (<55 years). Younger HFpEF patients may have different risk factors and outcomes compared to older patients. Despite having fewer comorbidities, younger HFpEF patients have a higher risk of cardiovascular death, particularly sudden death. Preventative measures early in life in high-risk individuals may reduce the overall burden of HFpEF [131-132].

Sex Differences in HFpEF

HFpEF exhibits sex differences in prevalence, risk factors, pathophysiology, and outcomes. Women predominate in HFpEF cases, outnumbering men by 2:1 [133]. While both sexes have similar lifetime HFpEF risk, women have a higher lifetime risk of HFpEF than HFrEF [134]. Women experienced less decline in HFpEF incidence over time compared to HFrEF [135]. Differences in risk factors and pathophysiological mechanisms contribute to these sex disparities [136]. Women are more prone to augmented arterial pressure (hypertension) due to smaller vasculature and poorer diastolic reserve, leading to greater arterial stiffness and more concentric LV hypertrophy [137]. Female-specific risk factors, such as Takotsubo cardiomyopathy and autoimmune disorders, trigger endothelial inflammation and microvascular dysfunction in HFpEF [138]. Despite similar prognosis, some treatments may benefit women more [139-140].

Treatment strategies

Overview of Current HFpEF Management Guidelines

Based on the guidelines recommended by the American Heart Association (AHA), the American College of Cardiology (ACC), and the ESC, the management of HFpEF typically includes complex and individual approaches [141-143]. All the patients should be encouraged to lead a healthy lifestyle with regular exercise, a balanced diet (e.g., Dietary Approaches to Stop Hypertension (DASH)) [144], and avoid smoking and alcohol overuse [142-144]. Patients need to have administered diuretics, such as loop or thiazide, that are helpful in fluid retention management and reduce symptoms [144-145]. Blood pressure has to be regularly controlled by the patients. They also should follow individualized pharmacotherapy based on angiotensin-converting enzyme (ACE) inhibitors, ARBs, or beta-blockers. Since HFpEF often coexists with other medical conditions like diabetes and CKD, managing these comorbidities helps to improve the patient’s quality of life and symptoms [143,145]. For heart rhythm and rate control, antiarrhythmics should be implemented. When AF coexists with HFpEF, anticoagulation therapy may be required [143-145].

Pharmacological Interventions and Their Limitations

There is no specific therapy proven to dramatically improve outcomes in HFpEF compared to HFrEF [146]. Management often involves treating comorbidities such as hypertension, diabetes, and obesity, as well as addressing lifestyle changes like diet and exercise [146-147]. Even though there is no strict pharmacological approach in HFpEF, management may include certain medications [147].

Diuretics may help reduce fluid retention and congestion, which alleviates symptoms like shortness of breath and edema [148-150]. Both loop diuretics and thiazides may be used [149-151]. Beta-blockers reduce the heart rate and decrease the blood’s overload on the heart, which can improve symptoms and possibly slow the progression of HFpEF [152]. Carvedilol and metoprolol are among the most commonly prescribed medications of this group [151-153]. ACE inhibitors or ARBs dilate blood vessels, reducing the blood’s overload on the heart and lowering blood pressure [154]. MRA reduces fluid retention and improves symptoms in patients (e.g., spironolactone, eplerenone) [155-156]. Calcium channel blockers may be used to manage high blood pressure. Amlodipine is a commonly used drug from this group [156]. Inotropic agents may be used to increase the heart’s contractility (e.g., digoxin). However, their use in HFpEF is limited due to the wide range of potential side effects [157-159].

Role of Lifestyle Modifications in HFpEF Management

Lifestyle modifications play a crucial role in HFpEF management. Such a change improves symptoms, enhances quality of life, reduces hospitalizations, and slows the progression of the condition [157]. HFpEF patients should adopt a heart-healthy diet, e.g., the DASH diet, which is based on fruits, vegetables, whole grains, lean proteins, and low-fat dairy products [157]. Limiting sodium and saturated fats is highly recommended. Reducing salt intake helps manage fluid retention, which is common in HFpEF [157-158].

Regular physical activity is also essential. Patients should engage in aerobic exercises, such as walking, swimming, or cycling. Exercise regimens should be tailored to individual capabilities and monitored by a healthcare professional [157]. Both dietary changes and exercise are of particular use in achieving and maintaining a healthy weight, as excess body weight can strain the heart and worsen symptoms [157]. Furthermore, patients should monitor fluid intake, since excessive fluid retention can lead to exacerbation of HFpEF symptoms. A strict recommendation on fluid restriction should be followed, especially if the patient experiences symptoms of fluid overload [157-158].

Smoking cessation is vital for overall cardiovascular health and is highly recommended for HFpEF patients [157-158]. They should also limit alcohol consumption since its intake can be detrimental to the heart, deteriorating liver function, and contributing to the edemas [157-158]. In cooperation with their healthcare professionals, patients should also focus on blood pressure control, diabetes, and stress management. Lifestyle modifications should be implemented in conjunction with medical approaches [157-159]. Patients' needs vary, and a personalized strategy based on regular follow-up appointments is essential to monitor HF progress and make necessary adjustments to the treatment plan.

Emerging therapies and their potential for HFpEF treatment

Several novel treatment strategies for HFpEF are being studied. SGLT2 inhibitors are widely used to treat type 2 diabetes and HFrEF, as they block transporters responsible for the reabsorption of glucose with Na+ from the proximal tubules [160-161]. Both dapagliflozin and empagliflozin can be of potential use in HFpEF [160-161]. SGLT2 reduces hospitalizations and cardiac remodeling [161]. Vericiguat is a soluble guanylate cyclase stimulator that has shown promise in the treatment of HFpEF [162]. The VICTORIA trial demonstrated a reduction in the risk of cardiovascular death and HF hospitalizations in patients with worsening HFpEF symptoms [162]. Angiotensin receptor/neprilysin inhibitor (ARNI) therapy such as sacubitril/valsartan has been approved for the treatment of HFrEF [163]. Ongoing research is exploring their potential benefits in HFpEF patients as well. Preliminary data suggests that ARNI therapy might enhance outcomes in some HFpEF patients [163-164].

Sildenafil, a PDE-5 inhibitor, used commonly in the treatment of erectile dysfunction and pulmonary hypertension has been found to improve exercise capacity and quality of life in some HFpEF patients, but needs further research [165]. Drugs like sildenafil have been also found to improve exercise capacity and quality of life in some HFpEF patients, but further research regarding its possible side effects and utility for disease management is needed [165]. Inorganic nitrates can be of potential use in enhancing exercise capacity and ameliorating HFpEF patients' quality of life [35,166]. Nitrates improve NO bioavailability, dilate vessels, and reduce the workload on the heart [35,167]. Cardiac amyloidosis is a condition that is mainly responsible for HFpEF progression [168-169]. There are some approaches based on tafamidis [169-171], which stabilizes transthyretin proteins and may potentially improve outcomes in patients’ amyloidosis [169-172].

Personalized Medicine Approaches Based on Patient Phenotypes

Personalized medicine approaches based on patient phenotypes in HFpEF aim to tailor treatment strategies to the specific needs of patients to improve outcomes and optimize care. Phenotyping and risk stratification are focused on involving the clinical, biochemical, and imaging parameters that categorize patients into subgroups with different pathophysiology of HF, comorbidity, and disease severity [35]. Risk stratification is particularly important in identifying high-risk patients who would benefit from more aggressive interventions [35,173].

Biomarker-guided therapies, such as BNP and NT-proBNP, are specific molecules found in the blood that provide valuable information about a patient’s health condition [35,173]. They could both be helpful in individualizing treatment plans and monitoring therapeutic responses [173]. Personalized therapies should be always designed individually by the healthcare professional. They have to include comorbidities management, tailored pharmacotherapy, exercise training programs, lifestyle modifications, and device-based therapies such as CRT and ICDs if needed [35,146-147].

Novel insights and advancements

Targeting Inflammation and Fibrosis Pathways As Potential Therapeutic Avenues

Chronic pro-inflammatory conditions can precipitate HFpEF [174]. Inflammation increases the levels of ICAM-1, VCAM-1, TFN-α, and IL-6, as well as increased oxidative stress and activation of eNOS, subsequently leading to oxidation of PKGIα [175]. The administration of empagliflozin resulted in a reduction of inflammation and oxidative stress in HFpEF, consequently enhancing the NO-sGC-cGMP pathway and PKGIα function through diminished PKGIα oxidation and polymerization, resulting in a reduction of detrimental cardiomyocyte stiffness [176]. Additionally, in the EMPA‐REG OUTCOME, empagliflozin reduced cardiovascular mortality only a few months after initiation of treatment [176].

An additional approach is enhancing the endothelial function in patients with HF which involves focusing on the generation of NO. ACE inhibitors, like quinaprilat, improve endothelial function in patients with HF by reducing the breakdown of bradykinin [177-178]. This facilitates endothelial production of NO, prostacyclin, and endothelium-derived hyperpolarizing factor [179-180]. Furthermore, the utilization of nitroglycerin as a direct source of NO has been employed to overcome the impaired NO-mediated endothelium relaxation in HF patients by triggering the relaxation of smooth muscle cells [181].

New Perspectives on Endothelial Function and Its Role in HFpEF

Recently, a new idea has emerged to address endothelial dysfunction, and this idea focuses on discovering eNOS enhancers [182]. The latest investigations managed to identify two compounds, AVE9488 and AVE3085, which are similar in low molecular weight and structure. They are able to stimulate eNOS transcription while inhibiting eNOS uncoupling [183] and improving capillary perfusion, thus showing ant ischemic and antihypertensive effects in rodent models of cardiovascular disease [184]. The results of these early-stage studies underscore the therapeutic potential of eNOS enhancers and propose the need for further exploration of these compounds [185].

The endothelium has many roles in regulating vascular tension by producing different substances that induce relaxation. These are vasodilator prostaglandins, NO, and endothelium-dependent hyperpolarization factors. In addition, the endothelium produces substances that contribute to vasoconstriction. This redundant mechanism mediated proper vascular tension [186-187]. In patients with HF, the endothelium-dependent vasodilatation is blunted, and this damage is shown in different vascular beds [172]. However, in HF, the significant involvement of compromised endothelial vasodilation can be explained by the influence of the equilibrium between the generation of NO and superoxide in the blood vessel system [188]. In HFpEF, metabolic risk factors generate inflammation and activation of the endothelium within the myocardial microvasculature. This process implicates heightened oxidative stress, eNOS uncoupling, diminished NO availability, and compromised GMP/protein kinase G signaling, resulting in dysfunction of the coronary microcirculation. These observations suggest that inflammatory changes within the myocardial microvasculature, prompted by metabolic coexisting conditions, may play a significant role in the development of HFpEF [189].

The role of precision medicine

Importance of Identifying Distinct HFpEF Subtypes

At present, there are four potential classification frameworks for HFpEF, which we will detail. The first classification is based on pathophysiology. The second is a clinical/etiologic classification. The third classification is according to the type of clinical presentation. The fourth one is the phenomics (or "phenomapping") of HFpEF [190]. It is important to recognize the heterogeneity of HFpEF, and categorizing patients into specific subtypes can create more targeted research and therapeutic interventions.

The heterogeneity of HFpEF has been identified as a prime reason why clinical trials have not yielded effective results in HFpEF patients, therefore, suggesting that a "one-size-fits-all" approach does not work in HFpEF [191]. A desirable HFpEF classification system would group individuals with similar pathophysiological traits, potentially leading to more consistent treatment responses [192]. Numerous studies have utilized machine learning techniques to pinpoint subgroups within HFpEF patients, classifying them into distinct clinical phenotypes and advocating for tailored treatments for each subgroup [193-195].

Tailoring Treatment and Challenges Along With Opportunities in Implementing Precision Medicine in HFpEF

Optimal treatment outcomes can be achieved by customizing interventions to individual patients who actively participate in their care and possess the skills to adhere to treatment plans. These patients experience improved survival rates and reduced hospital readmissions [196-198]. In personalized interventions, an individual's unique characteristics are evaluated, and the intervention is adjusted accordingly to enhance the pertinence of the treatment and generate more significant positive changes [199].

The presence of coexisting conditions significantly influences the outcomes of HFpEF. Managing conditions like hypertension, diabetes, and CKD requires customized interventions to mitigate their contributions to HFpEF development. For instance, optimizing blood pressure control has a favorable impact on cardiovascular health in HFpEF patients [200]. Therefore, tailoring pharmacological therapy to match patient needs is of utmost importance.

Challenges in implementing precision medicine in HFpEF are multifaceted. A significant hurdle is the heterogeneity of HFpEF patients. Thus, it is important to emphasize the need to define subgroups within HFpEF based on distinct phenotypic and molecular profiles to guide targeted interventions [190]. Another challenge is the lack of validated biomarkers that can be used to identify patients who are likely to respond to specific treatments [192]. In addition, there are challenges related to the cost and availability of precision medicine technologies, as well as the need for specialized training and expertise to interpret and apply the results of precision medicine tests [189].

Technological advancements, exemplified by high-throughput omics profiling, provide extensive insights into the molecular profiles of patients. Ongoing research is investigating the potential of transcriptomic and proteomic analyses in identifying unique pathways and potential therapeutic targets within specific HFpEF subgroups [201]. The benefit is that future treatment can be targeted on a molecular level to help treat patients with HFpEF along with patients suffering from any type of HF.

Future directions

Promising Areas for HFpEF Research and Investigation

Some studies have shown the GHRH receptor signaling pathway as a new molecular target against dysfunctional cardiac myocytes due to its effect on cardiomyocyte relaxation by phosphorylation and fibrosis. This opens the possibility of the use of GHRH agonists for the management of myocardial changes associated with HFpEF [202].

A study model done on a group of mice with hypertension-induced HFpEF showed that infusion of Apo a-I nanoparticles caused a significant reduction in cardiomyocyte and perivascular fibrosis and improved myocardial capillary density. This study looks very promising as it shows that MDCO-216 completely reverses cardiac dysfunction and proves to be an effective therapy in this model [203].

Another promising study done on a rat model with HFpEF showed improved cardiac function with the use of neuromodulation by the use of low-level transcutaneous vagal stimulation (LLTS). It has been shown that LLTS can improve diastolic dysfunction by decreasing LV inflammatory infiltration and fibrosis through pharmacological blockade of the α7nAchR. These results look quite promising and support the basis for the use of neuromodulation to treat selected patients with HFpEF for which further Randomized trials must be done [204].

Integrating New Findings Into Clinical Practice

Using investigations like speckle tracking echocardiography has proven useful in patients with HFpEF as an objective and quantitative measure by providing data about the myocardial mechanics, deformation, and strain parameters. It can be a tool for early indication and progression of HF. It can assess the impact of treatments in patients with HFpEF [204].

Physicians can incorporate the use of models by advanced machine learning techniques to better estimate the prognosis of patients with HFpEF. These models also consider the quality of life and health status data, which may not usually be there in a clinical encounter [205].

Potential Impact of Novel Therapeutic Interventions on HFpEF Outcomes

The role of estrogen and its mechanisms to prevent diastolic dysfunction of the heart looks promising for tackling HFpEF in post-menopausal women. Most of the proposed mechanisms involve a single E2 receptor in pathophysiology; therefore, it is plausible to develop therapy targeted toward this single receptor in order to avoid the non-cardiac effects of E2 therapy. This can have a huge impact on reducing the burden of HFpEF among post-menopausal women [205].

## Conclusions

HFpEF is a significant health problem with high mortality and morbidity. Its pathophysiology is known, yet, its clinical presentation is not specific, and its diagnosis is challenging. Diagnosis relies on the understanding of clinical symptoms, advanced imaging techniques such as CMR, semi-automatic cardiac image analysis, and segmentation software, frequently incorporating "AI" algorithms, metabolic imaging methods (phosphorus-31 cardiac magnetic resonance spectroscopy), and 11C-acetate PET, which provides valuable information about HFpEF. There has been a lot of focus on advancing the methods by which HFpEF is diagnosed, while treatment options remain the same. It's pertinent that future research establishes the efficacy of current therapy and explores novel treatment options. Further research is needed in the area of prevention of cardiomyocyte changes. The use of GnRH agonists in the management of HFpEF, infusion of Apo a-I nanoparticles, LLTS, and estrogen only in post-menopausal women are promising strategies to prevent diastolic dysfunction and HFpEF.
